# Biochar and conservation tillage affect the agronomic performance and fatty acid composition of *Nigella sativa* L. under both irrigated and dryland conditions

**DOI:** 10.1038/s41598-024-52425-5

**Published:** 2024-02-01

**Authors:** Hawre Kiani, Shiva Khalesro, Ali Mokhatssi-Bidgoli, Zahed Sharifi

**Affiliations:** 1 Department of Agronomy and Plant Breeding, Faculty of Agriculture, University of Kurdistan, Pasdaran Street, Sanandaj, 66177-15175 Iran; 2 Department of Agronomy, Faculty of Agriculture, Tarbiat Modares University, Tehran, 14115-336 Iran; 3 Department of Soil Science, Faculty of Agriculture, University of Kurdistan, Pasdaran Street, Sanandaj, 66177-15175 Iran

**Keywords:** Ecology, Plant sciences

## Abstract

Soils in arid and semi-arid regions like Iran have suffered greatly from low organic matter content and low water availability. Traditional tillage and the overuse of chemical fertilizers are accelerating the problems in the region. So, sensible and sustainable strategies such as conservation tillage and natural organic inputs are becoming increasingly important to enhance organic matter and humidity in the soil and grow high-quality crops in agroecosystems. Thus, in 2019 and 2020, a split-split plot arrangement within a randomized complete block design was conducted in Iran to assess the effects of irrigated conditions, tillage systems, and biochar on the aforementioned traits. There were two irrigation conditions (irrigated and dryland) as the main plots, three tillage methods (conventional, minimum, and no-tillage) as sub-plots, and two application rates for biochar (0 and 15 ton ha^−1^) as sub-sub plots. The findings indicated that biochar application enhanced grain yield across all tillage methods under both irrigation conditions. Biochar with minimum tillage improved oil yield by 23% and 29% compared to those that did not use biochar under the dryland and irrigated conditions, respectively. Moreover, oil yield was higher in 2020 than in 2019 for all tillage systems and biochar rates. The main components of *Nigella sativa* L. oil belong to linoleic, oleic, and palmitic acids. Minimum tillage with biochar under irrigated conditions in 2020 and no-tillage without biochar under dryland conditions in 2019 had the most (59%) and the least linoleic acid (53%), respectively. Conventional, minimum, and no-tillage with biochar in dryland conditions significantly increased linoleic acid by 2%, 3%, and 5% compared to those without biochar in 2020, respectively. In general, adopting biochar with minimum tillage produced the best outcomes for *Nigella sativa* L. yield, and grain oil quality under both irrigation conditions. It is recommended that farmers incorporate these practices to produce high-quality *Nigella sativa* L. in sustainable agricultural systems.

## Introduction

Soil organic matter (SOM) improves soil structure and properties such as soil density, porosity, infiltration, drainage, aeration, water-holding capacity, and resistance to erosion^1^. The SOM also supports microbial activity, buffers pH variations, and releases plant nutrients upon microbial mineralization^2^. A decline in SOM is a key facet of soil degradation, such as the loss of soil fertility and capacity to produce crops^3^. Thus, conservation tillage and soil amendment like biochar can improve SOM, especially in arid and semi-arid regions^4,5^. Compared to other amendment materials, biochar has the benefit of having a wide surface area and pore spaces, allowing it to absorb and retain water^6^.

Conservation tillage encompasses a range of practices that minimize soil disturbance and promote healthy ecosystems. Two common conservation tillage methods are minimum tillage and no-till, which offer numerous benefits for water quality, soil health, and the environment. These techniques support sustainable agriculture while preserving natural resources by reducing soil erosion, improving moisture retention, and supporting beneficial microorganisms. Also, it reduces fuel consumption and lowers greenhouse gas emissions^7^. So, conservation tillage is beneficial not only for the environment and the economy but also for crop productivity. For instance, He et al.^8^ reported that no-tillage significantly increased the grain yield of winter wheat (*Triticum aestivum* L.) and spring maize (*Zea mays* L.). Similarly, Kühling et al.^9^ found that no-tillage enhanced wheat yield and soil moisture under dryland conditions.

Biochar is one of the most environmentally friendly ideas that has been thought of in recent years^10^. Most of the world's interest in biochar comes from agricultural soils getting worse and water resources running out. Biochar reduces water consumption and improves soil properties under drought conditions. This natural input increases crop growth and nutrient uptake under water deficiency stress^11^. Mulcahy et al.^12^ found that water deficit stress had less harmful impact on tomato productivity when biochar was used.

*Nigella sativa* L. is a highly regarded medicinal and aromatic plant belonging to the Ranunculaceae family. It is a short-lived annual plant of tropical dicotyledon species are mainly grown in arid and semi-arid areas such as Saudi Arabia, Syria, Egypt, India, Turkey, Pakistan, and Iran^13^. It is also cultivated throughout the Mediterranean, Central Europe, and Western Asia^14^. In Iran, *Nigella sativa* L. is cultivated in various areas and widely used in people’s diets, and traditional medicine^15^. This plant also has high applications in modern pharmaceuticals as a blood glucose lowering, muscle relaxant, antibacterial, antiviral, antiepileptic, and anti-cancer agent^16,17^. Seeds contain fixed oil, protein, essential oil, and over a hundred phytochemical constituents such as alkaloids, tannins, resins, flavonol triglycosides, and saponins^18^. Majdalawieh et al.^19^ found that the grain of *Nigella sativa* L. has the highest amount of fixed oil, including linoleic and oleic acids.

The market size of *Nigella sativa* L. oil was 15 million USD in 2018, and a growth of 10 million USD by 2025 is predicted. The demand for grain oil is estimated at 700 tons for the nutraceutical, cosmetics, pharmaceuticals, and flavoring industries^20^. Thus, grain yield and oil improvement in *Nigella sativa* L. are very important.

Many research studies have underlined the useful effects of implementing conservation tillage and biochar on soil fertility and plant productivity. No study has evaluated the impact of these management factors on the morphological traits, grain and oil production, and fatty acid composition of *Nigella sativa* L. under dryland and irrigated conditions. Furthermore, there is a consensus that specific interactions between biochar, tillage, irrigation, and *Nigella sativa* L. must be considered, limiting the ability to make generalizations.

Therefore, this study aimed to discover the main effects and interactions of these agronomic management factors on the aforementioned traits of *Nigella sativa* L. The hypothesis was that by implementing conservation tillage and biochar application, soil physico-chemical attributes could be improved such as organic matter, cation exchange capacity, and water holding capacity, which would subsequently lead to enhanced yield and nutritional value of *Nigella sativa* L., especially under drought stress. This study aims to establish a solid theoretical framework for sustainable *Nigella sativa* L. production in soils where a lack of organic matter and water availability are the main constraints on crop growth.

## Material and methods

### Experimental conditions

**Figure 1:**
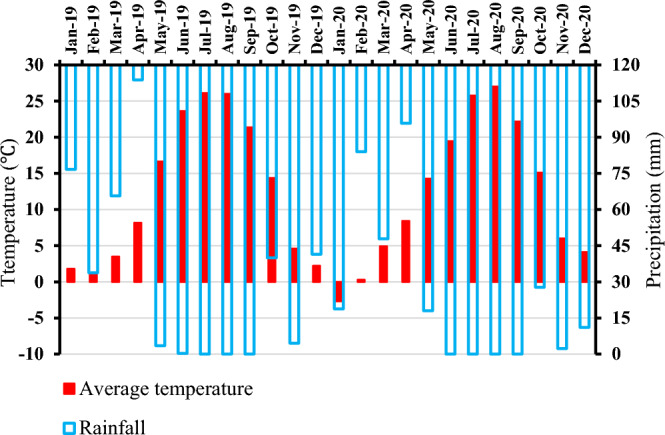
Monthly means temperature and total precipitation in the 2019 and 2020 growing seasons in the experimental area.

The field experiment was set up at the Research Field at the University of Kurdistan, which is in the northwestern part of Iran (35° 19′ N, 47° 18′ E), where a semi-arid climate prevails. The site elevation from mean sea level is 1866 m. The long-term mean annual participation and annual mean temperature of the area are 331 mm and 12 °C, respectively. Figure 1 illustrates the experimental period’s meteorological data. The experiment started in April 2019 and lasted on for two growing seasons until September 2020. Before the beginning of the trial, the field was planted with hairy vetch (*Vicia villosa* Roth.). Soil physiochemical characteristics from 0 to 30 depth in both years are presented in Table 1.

**Table 1:** Soil physicochemical characteristics of the experimental site.

Years	Soil texture	pH	EC (dS m^−1^)	O.C (%)	Total N (%)	Available P (mg kg^−1^)	Available K (mg kg^−1^)	Available Fe (mg kg^−1^)	Available Zn (mg kg^−1^)
2019	Clay-loam	7 ± 0.27	0.38 ± 0.02	0.41 ± 0.00	0.11 ± 0.03	10 ± 0.49	245 ± 5.11	8 ± 0.48	0.15 ± 0.00
2020	Clay-loam	7 ± 0.32	0.47 ± 0.01	0.63 ± 0.02	0.21 ± 0.01	11 ± 0.56	286 ± 5.84	8 ± 0.67	0.24 ± 0.01

Values are mean of three replicates ± SE.

### Biochar production

Cattle manure from the Husbandry Station (35° 25′ N, 47° 3′ E), Faculty of Agriculture, University of Kurdistan, Iran, was used to produce biochar. The precursor material was air-dried, crushed, and sieved through a 2 mm mesh. The powdered cattle manure was then pyrolyzed in an electrical furnace. Biochar was produced through the process of pyrolysis on cattle manure at a temperature of 600 ± 30 °C for four hours under oxygen-limited conditions. Table 2 is a listing of the characteristics of biochar.

**Table 2:** The chemical characteristics of biochar used in the experiment.

Total N (%)	P (mg kg^−1^)	K (mg kg^−1^)	Zn (mg kg^−1^)	Cu (mg kg^−1^)	Ca (mg kg^−1^)	Mn (mg kg^−1^)	Fe (mg kg^−1^)	Mg (mg kg^−1^)	CEC (cmolc kg^−1^)	EC (dS m^−1^)	pH
1 ± 0.03	2384 ± 7.11	18,227 ± 9.24	156 ± 4.68	27 ± 0.62	8476 ± 6.94	306 ± 5.47	1301 ± 6.25	1221 ± 5.06	83 ± 0.47	7 ± 0.53	9 ± 0.76

Values are mean of three replicates ± SE.

### Experimental details and agronomic data collection

A split-split plot arrangement within a randomized complete block design with three replications was used to set up the experiment. There were two irrigation conditions (irrigated and dryland) as the main plots, three tillage methods (conventional tillage, minimum tillage, and no-tillage) as sub-plots, and two application rates for biochar (0 and 15 ton ha^−1^) as sub-sub plots. The land was tilled to a depth of 15 cm for minimum tillage and 30 cm for conventional tillage. For conventional tillage, moldboard plowing and disking were employed, while chisel plowing was utilized for minimum tillage. Biochar was applied before sowing *Nigella sativa* L. In conventional and minimum tillage treatments, biochar was mixed with the soil. In the no-tillage treatments, biochar was spread on the soil surface.

*Nigella sativa* L. seeds (Semirom ecotype, Pakan Bazr Company, Isfahan, Iran) were planted by hand between 0.5 and 1 cm deep on April 12th and 14th, 2019 and 2020, respectively. Pakan Bazr Company is one of the top seed companies in Iran (https://www.akpsho.com/en/shopview/2652/Pakan+Bazr+Esfahan). Thus *Nigella sativa* seeds were purchased from it.

It was sown at a rate of 6 kg ha^−1^, and any excess plants were removed at the 4-leaf stage to ensure optimal density. The distances between main plots, sub-plots, and sub-sub plots were 5, 1.5, and 1 m, respectively. Each plot had eight rows, each 4 m long. The rows were spaced 30 cm apart, and the distance between plants situated in rows was 2 cm. So, the total area of the net plot was 9.6 m^2^, with dimensions of 4 m in length and 2.4 m in width.

Irrigation was initiated after planting, with the plots receiving their first irrigation right after sowing the seeds. From that point on, the plots were irrigated once a week until maturity using drip irrigation, following the customary practices of the region. In irrigated conditions, the plant was first irrigated on April 12th, 2019 and April 14th, 2020, and the last irrigation was applied on August 30th and 31st, 2019 and 2020, respectively. During the experiment, supplementary irrigation was applied three times (blooming, flowering, and grain formation) under dryland conditions. The amount of irrigation water was 1500 and 6290 m^3^ in the same volume for each irrigation under dryland and irrigated conditions, respectively.

No pesticides or chemical fertilizers were used in this experiment. Whenever necessary, weeding was performed manually. Agronomic traits like plant height, number of capsules per plant, number of grains per capsule, and 1000-grain weight were randomly measured from five plants at full maturity in each plot. The plant was harvested on August 30th and 26th, 2019 and 2020, under dryland conditions, and on September 15th and 16th, 2019 and 2020, under irrigated conditions. The grain yield was calculated based on a 2 m^2^ harvested area in the middle rows. The samples were dried in the shade and cool conditions for two weeks after harvest. Then the seeds weighted to calculate the grain yield.

### Grain oil extraction and fatty acid analysis

To get the fixed oil out of *Nigella sativa* L., the Soxhlet extraction method with *n*-hexane was used. The extracted oil was isolated through rotary liquid solvent evaporation^21^. Then fatty acid methyl esters were prepared for analysis by GC-FID. Gas chromatography (Agilent 7890A) was used to analyze fatty acid methyl esters. The GC equipment was the fused silica capillary column DB WAX (60 m × 0.25 i.d) with a 0.25 μm film thickness (Wilmington, DE, USA) and a flame ionization detector. The carrier gas was nitrogen. The oven temperature was set as follows: 5 min at 170 °C, then 4 °C min^−1^ to 190, held for 15 min at 190 °C. The temperatures of the injector and detector were both 260 °C. Fatty acid methyl esters were found by comparing their retention durations to those of pure standards (Sigma-Aldrich, St. Louis, MO).

### Data analysis

Using the UNIVARIATE procedure in SAS 9.3 (SAS Institute, Cary, NC, USA), the normality test of residuals was carried out before the analysis of variance (ANOVA) for each year. The Brown and Forsythe test was then used to see if the error mean squares were homogeneous between 2019 and 2020 for each trait. As a result, a combined ANOVA was done using the GLM (general linear model) procedure in SAS. When testing the main effects, two-way interactions, and three-way interactions in their respective *F*-tests, the main, sub, and sub-sub plot errors are used as the error terms.

The means were separated based on priority from the three-way interaction to the main effects by the LSD (least significant difference) test at *p* ≤ 0.05. A power analysis was performed to determine the number of replications required to achieve a significance level of 0.05 with a power greater than 0.8^22^. Principal components analysis was conducted using the Microsoft Excel XLSTAT program (Version 2019.2.2.59614).

### Ethics statement

All the method was complied with relevant institutional, national, and international guidelines.

## Results

### Plant height and yield components

The significant main effects of tillage and biochar were seen in the plant height, number of capsules per plant, number of grains per capsule, and 1000-grain weight (Table 3). Except for the number of grains per capsule, irrigation significantly impacted the other yield components. Plant height and the number of capsules per plant were affected by irrigation × tillage and irrigation × biochar two-way interactions. In addition, irrigation × tillage × biochar had a significant three-way interaction for the yield components (Table 3).

**Table 3:** A combined analysis of variance of *Nigella sativa* L. characteristics affected by biochar (B) and tillage (T) under different irrigation (I) conditions in the 2019 and 2020 years (Y).

Source of variation	DF	Mean squares						
		Plant height	Capsule number per plant	Grain number per capsule	1000 grain weight	Grain yield	Grain oil content	Oil yield
Y	1	35ns	6ns	203ns	0.162ns	714ns	0.054*	2,819,571**
Replication (Y)	4	6	44	1138	0.070	1621	0.003	11,580
I	1	2980**	3208**	1997ns	2.25**	332,891**	127**	7,315,088**
Y × I	1	10ns	6ns	17ns	0.014ns	443ns	0.013ns	2,774,735**
Replication × I (Y)	4	32	51	983	0.105	1665	0.005	32,940
T	2	437**	28**	1105**	0.044*	20,966**	4**	259,876**
I × T	2	81**	24**	53ns	0.018ns	1737**	0.676**	79122ns
Y × T	2	0.254ns	0.031ns	0.114ns	0.00001ns	11ns	0.005ns	44960ns
Y × I × T	2	0.034ns	0.017ns	0.366ns	0.000002ns	4ns	0.005ns	43716ns
Replication × T (Y × I)	16	9	0.48	72	0.007	88	0.005	523
B	1	409**	20**	538*	0.276**	13,601**	55**	32,212**
I × B	1	40**	68**	18ns	0.011ns	2790*	7**	3111ns
T × B	2	10ns	2*	141ns	0.026ns	1025ns	0.017ns	11733ns
I × T × B	2	6ns	2*	241*	0.064*	1505*	0.056**	29,091*
Y × B	1	0.151ns	0.034ns	0.320ns	0.00005ns	8ns	0.026ns	177ns
Y × I × B	1	0.131ns	0.046ns	0.271ns	0.0002ns	7ns	0.020ns	158ns
Y × T × B	2	0.016ns	0.001ns	0.079ns	0.000004ns	0.558ns	0.014ns	29,516*
Y × I × T × B	2	0.004ns	0.004ns	0.032ns	0.00003ns	0.774ns	0.001ns	723ns
Error	24	4	0.69	81	0.015	428	0.007	7231
CV (%)		6.3	6.8	13.4	5.4	17.1	2.3	19.4

ns, *, and **p > 0.05, *p* ≤ 0.05, and *p* ≤ 0.01, respectively.

**Figure 2:**
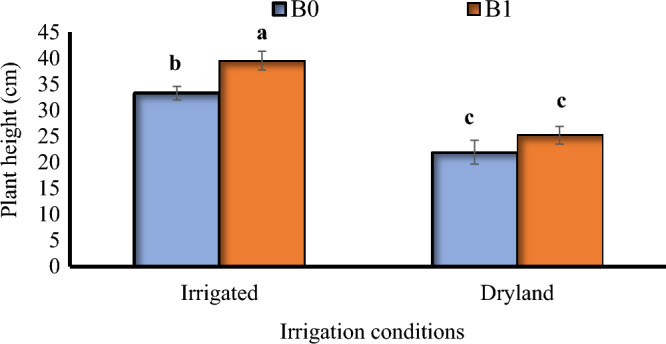
The interaction between biocharand irrigation conditions on the plant height of *Nigella sativa* L. B0, B1 = 0 and 15 ton ha^−1^ biochar. The same letters above the bars are not significantly different using the LSD test (*p* ≤ 0.05).

**Figure 3:**
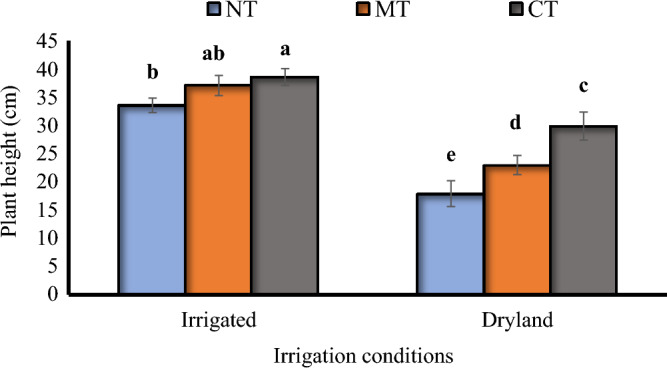
The interaction between tillage and irrigation conditions on the plant height of *Nigella sativa* L. *NT* no-tillage, *MT* minimum tillage, *CT* conventional tillage. The same letters above the bars are not significantly different using the LSD test (*p* ≤ 0.05).

The biochar treatment in the irrigated plots had the tallest plants with an average of 39 cm, and the dryland treatments without biochar had the shortest plants with an average of 21 cm (Fig. 2). There were significant differences between tillage systems in both irrigation conditions (Fig. 3). Conventional tillage in irrigated conditions led to the tallest plants, but there wasn't a significant difference between that and minimum tillage in the same conditions. Compared to minimum tillage and no-tillage on dryland conditions, conventional tillage made plants grow 23% and 40% taller, respectively (Fig. 3).

Biochar enhanced capsules number per plant in all of the tillage systems in both irrigation conditions (Table 4). Adding 15 ton of biochar ha^-1^ to the irrigated plots with conventional, minimum, and no-tillage treatments increased this property by 6, 8, and 7%, respectively, compared to the control with no biochar. These values were 9, 23, and 11% in dryland conditions (Table 4). In irrigated conditions, minimum tillage and biochar application led to the most number of grains per capsule (81), while conventional tillage led to the most number of grains per capsule (76) in dryland conditions (Table 4). In irrigated conditions, there were no significant differences in 1000-grain weight between the different treatments. The main difference was between irrigated and dryland conditions (Table 4).

**Table 4:** The three-way interaction of biochar, tillage, and irrigation conditions on the yield components, oil content, and oil yield in *Nigella sativa* L.

	Capsule number per plant	Grain number per capsule	1000 grain weight (g)	Grain yield (kg ha^−1^)	Oil content (%)	Oil yield (kg ha^−1^)
Irrigated						
NT						
B0	8 ± 0.31d	62 ± 3.14ce	2.5 ± 0.31ab	921 ± 5.11d	28 ± 0.49cd	258 ± 11.32f
B1	9 ± 0.38c	65 ± 3.81ce	2.6 ± 0.28a	1078 ± 8.43c	30 ± 0.35acd	3298.10d
MT						
B0	9 ± 0.42bc	67 ± 4.22c	2.5 ± 0.22ab	1067 ± 7.10c	29 ± 0.34bc	312 ± 7.80e
B1	10 ± 0.32ab	81 ± 3.29a	2.4 ± 0.32ab	1412 ± 9.42ab	31 ± 0.39a	444 ± 9.15b
CT						
B0	10 ± 0.47ab	77 ± 23b	2.3 ± 0.27abc	1264 ± 8.16b	28 ± 0.26cd	360 ± 6.15c
B1	11 ± 0.45a	79 ± 2.14ab	2.5 ± 0.30ab	1497 ± 9.70a	30 ± 0.31ab	463 ± 7.20a
Dryland						
NT						
B0	5 ± 0.19 h	57 ± 1.98ef	2.1 ± 0.19cd	404 ± 6.15h	26 ± 0.17g	128 ± 5.55k
B1	5 ± 0.21 g	54 ± 2.18f	2.1 ± 0.21bcd	672 ± 5.64f	27 ± 0.18ef	148 ± 4.20j
MT						
B0	5 ± 0.25gh	59 ± 2.50e	2.0 ± 0.25d	447 ± 5.89h	26 ± 0.14fg	161 ± 3.90i
B1	7 ± 0.24ef	62 ± 2.42ce	2.2 ± 0.24bcd	632 ± 7.21fg	28 ± 0.21cd	210 ± 4.80h
CT						
B0	6 ± 0.26f	61 ± 2.63ce	2.0 ± 0.26d	582 ± 5.34g	26 ± 0.14g	199 ± 3.22h
B1	7 ± 0.23e	76 ± 2.31b	2.2 ± 0.23bc	809 ± 6.87e	27 ± 0.25de	241 ± 5.85g

The same letters above the columns are not significantly different using the LSD test (*p* ≤ 0.05). Values are mean of three replicates ± SE.

### Grain yield

The main effects of irrigation, tillage, and biochar were significant on the grain yield (Table 3). The two-way interaction between irrigation and biochar and the three-way interaction of irrigation × tillage × biochar had a significant impact on the grain yield (Table 3). Biochar increased grain yield for each tillage system in both irrigation conditions (Table 4). In both irrigated and dryland conditions, conventional, minimum, and no-tillage with biochar increased grain yield by 15, 24, 14, 28, 29 and 39%, respectively, compared to the same tillage systems without biochar.

The best grain yield came from both conventional and minimum tillage that was treated with biochar and irrigated. No-tillage treatment with biochar was in a similar group to minimum tillage without biochar in irrigated conditions. The highest grain yield (80 g m^−2^) came from conventional tillage with biochar and the lowest (40 g m^−2^) came from no-tillage without biochar in dryland conditions. There were no significant differences between minimum tillage with biochar and conventional tillage without biochar under dryland conditions. Also, when biochar was used in dryland conditions, there were no significant differences between no-tillage and minimum tillage (Table 4).

### Grain oil content and oil yield

Both the grain oil content and oil yield were significantly impacted by the year (Table 3). The main impacts of all factors were significant on these characteristics. Besides, the three-way interaction of irrigation, tillage, and biochar was significant for both traits (Table 3). The most grain oil in irrigated (31%) and dryland (28%) conditions was made when there was the least amount of soil disturbance and biochar were used (Table 4). Biochar enhanced grain oil content in each tillage system and each condition (Table 4).

In irrigated conditions, conventional tillage with biochar made the most oil yield, while in dryland conditions, no-tillage without biochar made the least oil yield (Table 4). Conventional tillage with biochar increased oil yield by 4 and 28% in irrigated conditions, and 13 and 38% in dryland conditions, which is significantly more than minimum and no-tillage with biochar (Table 4). Conventional, minimum, and no-tillage with biochar increased oil yield by 22, 29, and 21 in irrigated conditions, and 17, 23, and 13% in dryland conditions, respectively, compared to those without biochar.

**Figure 4:**
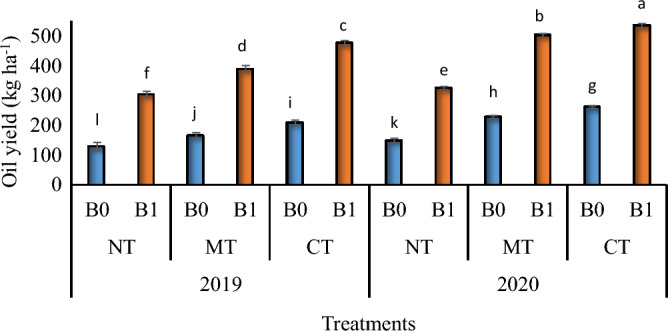
The interaction between biochar and tillage on the oil yield of *Nigella sativa* L. in 2019 and 2020. B0, B1 = 0 and 15 ton ha^−1^ biochar *NT* no-tillage, *MT* minimum tillage, *CT* conventional tillage. Thesameletters above the bars are not significantly different using the LSD test (*p* ≤ 0.05).

The important point here is that minimum tillage with biochar had a higher oil yield compared to conventional tillage without biochar in irrigated conditions, and there were no significant differences between those treatments in dryland conditions (Table 4). Moreover, for all tillage systems and biochar rates, oil yield was higher in 2020 than in 2019 (Fig. 4). Also, biochar application significantly increased oil yield in both years compared to control treatments (Fig. 4).

### Monounsaturated fatty acids

Table 5 shows that irrigation, tillage, and biochar had a significant effect on all of the monounsaturated fatty acids (MUFA), like palmitoleic, oleic, and eicosenoic acids. All MUFA were affected by year × tillage × biochar and year × irrigation × tillage × biochar interactions (Table 5).

**Table 5:** A combined analysis of variance of *Nigella sativa* L. fatty acids affected by biochar (B) and tillage (T) under different irrigation (I) conditions in the 2019 and 2020 years (Y).

Source of variation	DF	Mean squares									
		Unsaturated						Saturated			
		Monounsaturated			Polyunsaturated						
		Palmitoleic (16:1)	Oleic (18:1)	Eicosenoic (20:1)	Linoleic (18:2)	Linolenic (18:3)	Eicosadienoic (20:2)	Myristic (14:0)	Palmitic (16:0)	Stearic (18:0)	Arachidic (20:0)
Y	1	0.019ns	1*	1*	1ns	16**	0.751ns	0.002ns	0.793ns	0.139**	2**
Replication (Y)	4	0.058	0.115	0.138	4	4.34	0.255	0.027	0.205	0.0001	0.030
I	1	0.297*	6**	2**	17**	17**	0.952ns	0.006ns	0.835**	2**	3**
Y × I	1	0.0004ns	0.561*	1*	4*	4*	0.069ns	0.737*	0.225*	0.001ns	2**
Replication × I (Y)	4	0.026	0.036	0.041	0.58	0.58	0.19	0.066	0.027	0.002*	0.016
T	2	0.239**	0.982**	0.981**	4*	4*	0.203ns	0.333**	0.210*	0.13**	0.481**
I × T	2	0.333**	0.297**	0.035ns	8**	8**	0.059ns	0.089ns	0.385**	0.27**	0.609**
Y × T	2	0.168**	0.532**	0.536**	6*	6**	0.023ns	0.051ns	0.327**	0.19**	0.775**
Y × I × T	2	0.369**	0.304**	0.377*	2ns	2ns	0.052ns	0.358**	0.107ns	0.117**	0.735**
Replication × T (Y × I)	16	0.011	0.021	0.069	0.84	0.84	0.081	0.033	0.039	0.002	0.016
B	1	0.699**	3**	3**	10**	10*	0.137ns	0.819**	0.513*	0.60**	3**
I × B	1	0.552**	0.261*	2**	3ns	3ns	0.337ns	0.014ns	0.181ns	0.30**	3**
T × B	2	0.163*	0.329**	0.322*	5ns	5ns	0.348ns	0.321**	0.273ns	0.074**	0.522**
I × T × B	2	0.206**	0.319**	0.083ns	3ns	3ns	0.662ns	0.082ns	0.146ns	0.021**	0.562**
Y × B	1	0.005ns	0.332**	1*	1ns	1ns	0.0001ns	0.001ns	0.087ns	0.039**	2**
Y × I × B	1	0.138ns	2**	3**	9*	9*	0.262ns	0.611**	0.469*	0.032**	2**
Y × T × B	2	0.318**	0.285**	0.496**	7*	7*	0.011ns	0.061ns	0.349*	0.05**	0.823**
Y × I × T × B	2	0.352**	0.304**	1*	9*	9*	0.039ns	0.513**	0.446*	0.05**	0.694**
Error	24	0.034	0.041	0.069	1	1	0.21	0.036	0.093	0.002	0.018
CV (%)		11.9	1.8	14.6	2.4	2.6	14.4	19.2	2.5	1.8	16.5

ns, *, and **: p > 0.05, *p* ≤ 0.05, and *p* ≤ 0.01, respectively.

In both years, when the plots were tilled to minimum tillage with biochar, the grain oil had the most palmitoleic acid. In dryland conditions, the best way to treat this fatty acid in 2019 was with conventional tillage and biochar. In 2020, the best way to treat it was with minimum tillage and biochar (Table 6).

**Table 6:** The concentrations of *Nigella sativa* L. fatty acids (%) affected by biochar and tillage under irrigated and dryland conditions in 2019 and 2020.

	Palmitoleic (16:1)	Oleic (18:1)	Eicosenoic (20:1)	Linoleic (18:2)	Linolenic (18:3)	Myristic (14:0)	Palmitic (16:0)	Stearic (18:0)	Arachidic (20:0)
2019									
Irrigated									
NT									
B0	0.29 ± 0.03de	21.5 ± 0.17ab	–	57.0 ± 0.2d	–	0.21 ± 0.014e	12.2 ± 0.13bc	3.0 ± 0.01de	–
B1	0.52 ± 0.03b	21.6 ± 0.16ab	–	57.2 ± 0.3cd	–	0.14 ± 0.008gh	11. 9 ± 0.12ef	2.7 ± 0.01hi	–
MT									
B0	0.32 ± 0.03cd	21.8 ± 0.14a	–	57.1 ± 0.3cd	–	0.15 ± 0.01g	12.1 ± 0.13cd	2.9 ± 0.01efg	–
B1	0.56 ± 0.03a	21. 9 ± 0.14a	–	58.3 ± 0.3ab	0.44 ± 0.01c	0.13 ± 0.01h	11.5 ± 0.13gh	2.5 ± 0.03i	–
CT									
B0	0.37 ± 0.03c	21.5 ± 0.17ab	–	57.4 ± 0.2c	–	0.3 ± 0.02d	12.0 ± 0.14cde	2.9 ± 0.02e–g	–
B1	0.55 ± 0.03ab	21.6 ± 0.15a	–	58.5 ± 0.3ab	0.2 ± 0.02e	0.12 ± 0.01i	11.4 ± 0.13h	2.5 ± 0.02i	–
Dryland									
NT									
B0	0.13 ± 0.03h	19.2 ± 0.31d	2 ± 0.17a	53.1 ± 0.4h	–	0.39 ± 0.01c	12.6 ± 0.13a	3.5 ± 0.03a	3 ± 0.08a
B1	0.19 ± 0.03fg	21.3 ± 0.19b	0.54 ± 0.11d	55.7 ± 0.1ef	–	0.15 ± 0.01gh	12.2 ± 0.14bcd	3.0 ± 0.01ef	0.06 ± 0.01g
MT									
B0	0.16 ± 0.03h	20.9 ± 0.25bc	0.85 ± 0.1c	55.4 ± 0.3fg	–	0.52 ± 0.01a	12.4 ± 0.13ab	3.2 ± 0.06b	1 ± 0.05b
B1	0.28 ± 0.03f	21.5 ± 0.16ab	0.26 ± 0.08e	57.1 ± 0.2cd	–	0.17 ± 0.01fg	12.1 ± 0.13cd	2.9 ± 0.03e–g	–
CT									
B0	0.17 ± 0.03gh	20.8 ± 0.32c	1 ± 0.09b	55.6 ± 0.2ef	–	0.44 ± 0.01b	12.3 ± 0.13bc	3.1 ± 0.04bc	0.56 ± 0.09c
B1	0.37 ± 0.03de	21.4 ± 0.18ab	0.16 ± 0.08f	56.9 ± 0.2d	0.16 ± 0.01e	0.17 ± 0.02fg	12.1 ± 0.14cde	2.9 ± 0.01e–g	–
2020									
Irrigated									
NT									
B0	0.27 ± 0.03c	21.3 ± 0.18b	–	57.8 ± 0.3c	–	0.16 ± 0.02g	12.2 ± 0.15bc	3.0 ± 0.01ef	–
B1	0.46 ± 0.04b	21.9 ± 0.13a	–	58 ± 0.2bc	2.58 ± 0.05a	0.16 ± 0.02g	11.9 ± 0.14efg	2.7 ± 0.01hi	–
MT									
B0	0.30 ± 0.03de	21.5 ± 0.16ab	–	57.0 ± 0.2d	–	0.2 ± 0.02ef	12.0 ± 0.11def	2.7 ± 0.01hi	–
B1	0.60 ± 0.04a	22.1 ± 0.12a	–	59.0 ± 0.3a	0.77 ± 0.02b	0.14 ± 0.02h	11.6 ± 0.14fg	2.6 ± 0.01jk	–
CT									
B0	0.30 ± 0.03de	21.4 ± 0.17ab	–	57.5 ± 0.3c	0.13 ± 0.01e	0.15 ± 0.03g	12.0 ± 0.15de	2.8 ± 0.01gh	–
B1	0.54 ± 0.04ab	21.9 ± 0.12a	–	58.8 ± 0.4a	0.75 ± 0.03b	0.12 ± 0.02i	11.6 ± 0.14fg	2.7 ± 0.01h–j	–
Dryland									
NT									
B0	0.14 ± 0.04gh	21.2 ± 0.21b	0.45 ± 0.13de	53.8 ± 0.3gh	–	0.2 ± 0.02e	12.5 ± 0.14ab	3.2 ± 0.01bc	0.29 ± 0.01d
B1	0.27 ± 0.03e	21.3 ± 0.18b	0.43 ± 0.19de	56.9 ± 0.1d	0.18 ± 0.01e	0.16 ± 0.02ef	12.1 ± 0.15cd	2.9 ± 0.01e–g	–
MT									
B0	0.17 ± 0.03fg	21.3 ± 0.18b	0.35 ± 0.14e	55.6 ± 0.2ef	–	0.22 ± 0.03e	12.3 ± 0.14bc	3.1 ± 0.01cde	0.15 ± 0.01e
B1	0.33 ± 0.0cd	21.5 ± 0.16ab	0.09 ± 0.08g	57.7 ± 0.3c	0.29 ± 0.01d	0.15 ± 0.02g	12.0 ± 0.14de	2.8 ± 0.01gh	–
CT									
B0	0.16 ± 0.03fg	21.2 ± 0.2b	0.28 ± 0.1e	56.1 ± 0.2e	–	0.17 ± 0.02fg	12.2 ± 0.14bcd	2.9 ± 0.03e–g	0.11 ± 0.01f
B1	0.31 ± 0.04cde	21.2 ± 0.19b	0.16 ± 0.1f	57.6 ± 0.4c	0.07 ± 0.01f	0.15 ± 0.02gh	12.0 ± 0.15de	2.8 ± .02gh	–

The same letters above the columns are not significantly different using the LSD test (*p* ≤ 0.05). Values are mean of three replicates ± SE.

Minimum tillage with biochar in irrigated conditions in 2020 had the most oleic acid, at 22%, and no-tillage without biochar in dryland conditions in 2019 had the least oleic acid, at 19%. Minimum tillage in dryland conditions had no significant difference with the better group in both years (Table 6). Eicosenoic acid only exists in dryland conditions in both years. Using biochar decreased this fatty acid. In both years, there was less eicosenoic acid in minimum and conventional tillage systems than in no-tillage. Also, the amount of this fatty acid was lower in 2020 than in 2019 (Table 6).

### Polyunsaturated fatty acids

Linoleic and linolenic acids were significantly affected by irrigation, tillage, and biochar. Besides, these fatty acids were influenced by year × irrigation × biochar, year × tillage × biochar, and year × irrigation × tillage × biochar interactions (Table 5). Eicosadienoic acid (1–4%) was not significantly affected by the studied treatments (Table 5).

The minimum tillage with biochar in irrigated conditions in 2020 and no-tillage without biochar in dryland conditions in 2019 had the most linoleic acid (59%) and the least (53%), respectively. Conventional, minimum, and no-tillage with biochar in irrigated and dryland conditions significantly increased linoleic acid by 1, 2, 0.35, 2, 2, and 4% in comparison with those without biochar in 2019, respectively. These values were 2, 3, 0.35, 2, 3, and 5% in 2020 (Table 6). Linolenic acid was not observed in all treatments. There was no way to tell if it had anything to do with it, but it was seen in all treatments with biochar in 2020 (Table 6).

### Saturated fatty acids

Except for myristic acid, irrigation had a significant effect on saturated fatty acids (SFA), such as palmitic, stearic, and arachidic acids (Table 5). The main effects of tillage and biochar and the four-way interaction of year × irrigation × tillage × biochar were significant on all SFA (Table 5). Biochar decreased myristic, palmitic, and stearic acids in both conditions in 2019 and 2020 (Table 6).

Under dryland conditions in 2019, minimum tillage without biochar had the highest amount of myristic acid (0.52%), while no-tillage without biochar had the highest amounts of palmitic acid (12%) and stearic acid (3%). In 2019, conventional tillage treated with biochar had the lowest amounts of myristic (0.12%), palmitic (11%), and stearic acids (3%) in grain oil under irrigated conditions (Table 6). Arachidic acid only existed in some dryland treatments. In 2019, Table 6 shows that no-tillage without biochar had the highest amount of arachidic acid (3%), while no-tillage with biochar had the lowest amount (0.06%).

### Principal components analysis

**Figure 5:**
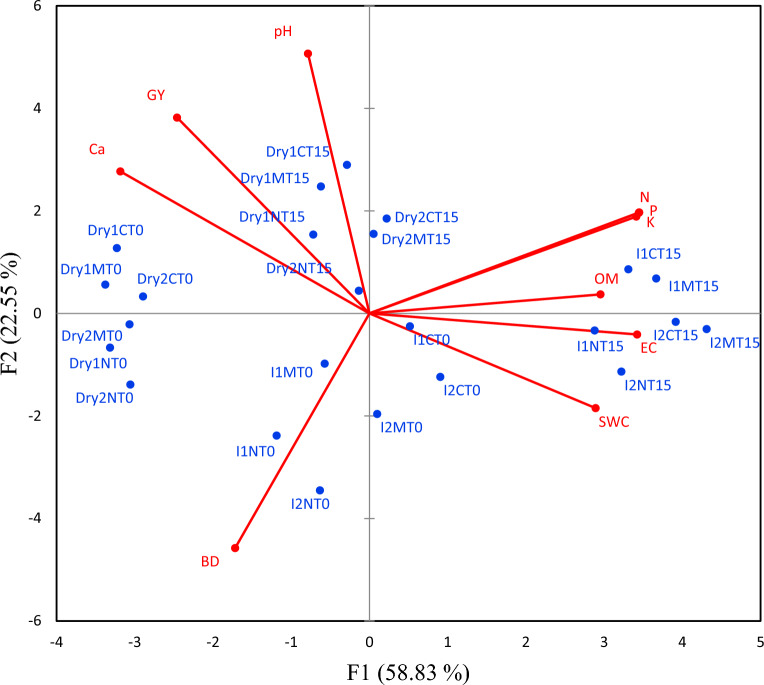
The biplots represent a principal components analysis of irrigation conditions [irrigated (I) and dryland (dry)], tillage (NT, MT, and CT), and biochar (0 and 15 ton ha^−1^) on the grain yield (GY) of *Nigella sativa* and on the electrical conductivity (EC), pH, soil water content (SWC), bulk density (BD), organic matter (OM), P, Ca, N, and K of soil in 2019 and 2020 (years 1 and 2).

A principal component biplot was used in this study to look at the links between the different treatment combinations and the different traits that were measured in soil and N. sativa cultivation (Fig. 5). The principal component analysis (PCA) showed that the top two principal components (PCs) explained over 80% of the overall variance in the data. This suggests that these two components account for the majority of the variability in the dataset. Under dryland conditions (Fig. 5), biochar had the main impact on grain yield. The effect of the mentioned treatments was higher in the first year compared to the second year due to the higher rainfall in the first year. No tillage systems without biochar had a main impact on the bulk density due to the lower organic matter in these treatments. Irrigated treatments had an effect on the soil water humidity, as one may expect. Moreover, NPK availability and organic matter influenced by biochar application in irrigated treatments consequently affect soil EC. In contrast, biochar in dryland treatments was more effective on grain yields due to the higher water use efficiency. In general, biochar has a good role in low-fertility soil.

## Discussion

Drought stress is the major limiting factor for crop production in farming systems^23^. So, soil amendments and tillage systems that can reduce their negative impacts are useful and practical approaches, particularly in arid and semi-arid regions. In this study, plants were shorter when they were in a dryland condition than when in an irrigated condition. As a result of this environmental stress, plants frequently reduce their growth rate. They must adjust their focus from growing leaves and stems to produce molecules and compounds that can protect them from the effects of drought. Producing a variety of protective compounds, including antioxidants, osmoprotectants, soluble proteins, and proline, is one of the primary strategies plants use to survive drought^24^. These substances reduce cellular damage caused by dehydration, stabilize cell membranes, and protect against oxidative stress. By reallocating resources to these protective measures, plants can better withstand the rigors of a drought^25^.

Also, a decrease in cell division and turgor potential due to drought can result in stunted plant growth. This result is the same as other studies found, which showed that drought stress made *Ammi visnaga* L.^26^ and *Nigella sativa* L. plants shorter^14^.

Based on the results, using biochar increased the plant's height. Nitrogen is a vital nutrient for supporting plant growth. Applying biochar in soil may encourage the immobilization of bioavailable nitrogen and phosphorus, which may otherwise be lost through leaching or emissions into the environment^27^. In addition, biochar has a high C:N ratio, which favors nitrogen immobilization in the soil. Thus, the boosted adsorption capacity of biochar played an essential role in maintaining soil nitrogen^28^. Indeed, biochar could act as a slow-release fertilizer due to its nutrient retention, greenhouse mitigation, and carbon sequestration traits^29^. This, in turn, enhances crop growth and productivity under normal conditions and soils subject to abiotic stress^30^.

In line with this result, other studies showed that biochar-made French bean (*Phazeolus vulgaris* L.)^31^ and basil (*Ocimum basilicum* L.) grow taller^32^. Tillage significantly increased plant height under both irrigation conditions. Because the roots don't spread out as much in a no-till system, you can probably expect the plants to grow less and get shorter. Previous showed that conventional tillage led to the tallest wheat and corn plants and that no-tillage led to the shortest wheat and corn plants^33^, which is in line with the results of this study.

Yield components and grain yield were reduced in dryland conditions compared to irrigated conditions. *Nigella sativa* L. has a determinate growth habit. So, it will move quickly from the vegetative to the reproductive phase if the drought stress gets worse^34^. When biochar and minimum tillage are used together, they can probably improve grain yield by making the soil less compact, adding more organic matter, and letting roots grow deeper. Reducing moisture loss has allowed the roots to use nitrogen and other nutrients from biochar.

Moreover, biochar utilization positively affects soil microbial activity due to its large specific surface area, porosity, functional groups, minerals, and surface volatile organic compounds (VOCs). VOCs can induce toxicity in microbial soil pathogens as microbial inhibitors, thereby advantaging plant growth^35^. These alterations could improve soil condition and plant-soil water relations, enhance the nutrient cycle, reduce nutrient leaching, form labile carbon compounds for microbial growth, enhance moisture retention, increase photosynthesis rate, and accelerate the plant growth mechanism^36^. This explains biochar's role in water-soluble nutrient availability and grain yield enhancement, especially under drought stress. These findings are in agreement with. Głodowska et al.^37^ and Rab et al.^38^ pointed out that biochar significantly increased the yield components and grain yield of soybean (*Glycine max* (L.) Merr.) and mung bean (*Vigna Radiata* (L.) Wilczek.). Abdullah et al.^39^ reported that conventional tillage without residue decreased grain yield by 24% compared to minimum tillage with residue.

In this study, biochar enhanced oil content and yield in both irrigation conditions. In 2020, oil content and yield increased compared to 2019. It seems that the effects of biochar and minimum tillage added up to positively affect oil content and yield in the second year, when soil conditions and nutrient availability were better. Drought stress at the late growth stage, decreased photosynthesis, and shortened seed filling time explain the low oil content in dryland compared to irrigated conditions.

Previous studies^40,41^ showed a significant drop in the amount of oil in soybean, safflower (*Carthamus tinctorius* L.), and *Nigella sativa* L. when drought stressed them. One reason for the oil increment is the rich content of biochar in terms of macro and micronutrients such as phosphorus, calcium, iron, copper, zinc, and especially potassium (Table 1), which improves drought stress alleviation, enhances carbohydrate metabolism, and influences oil content. In addition, any factors that enhance grain yield can increase oil yield due to the positive correlation between oil yield with grain oil content and grain yield. Suppadit et al.^42^ reported the positive effect of biochar on the grain oil content of soybean.

The main components of *Nigella sativa* L. oil belong to linoleic, oleic, and palmitic acids (Table 4). This finding is consistent with previous research, which identified these fatty acids as the major components in *Nigella sativa* L. oil^43,44^. Linoleic acid is one of the essential fatty acids because it cannot be synthesized by humans. Fatty acids had different reactions to the studied treatments. Abiotic stresses, including water and nutrients, affect the oil yield and fatty acid composition of medicinal plants^45^. Some UFA, like palmitoleic, oleic, and linoleic acids, in contrast to SFA, were lower in both years in dryland conditions than in irrigated conditions.

It may be attributed to the decreased enzyme activity of oleate desaturase. High temperatures strongly reduced the mentioned enzyme activity^46^. Therefore, the higher temperature during the grain development stage in dryland conditions resulted in lower UFA than irrigated conditions. In agreement with these results, Amiri-Darban et al.^47^ pointed out drought stress reduced oleic, linoleic, and linolenic acids and increased eicosenoic acid in camelina (*Camelina sativa* L. Crantz.). Biochar and tillage, especially minimum tillage, increased palmitoleic, oleic, and linoleic acids and decreased myristic, palmitic, and stearic acids. Organic fertilizers can affect soil biochemical characteristics and macro- and micro-nutrients uptake by plants. It may have influenced the enzymes and genes involved in the biosynthesis of fatty acids^48^.

Thus, it can be said that biochar compounds can affect the biosynthesis and chain reaction of fatty acids. Similarly, previous studies showed that biochar increased palmitoleic acid and decreased palmitic acid in corn^49^, and minimum tillage positively affected UFA^50^. Gavili et al.^51^ stated that the large specific surface area, porous structure, large specific surface area, and high cation exchange capacity of biochar make the soils’ properties better. Biochar seems to provide moisture and essential nutrients for high-quality oil production. It also seems to lessen the negative impacts of drought stress. It can be said that biochar and minimum tillage have synergistic relationships. It is probably true that minimum tillage promotes the positive effects of biochar.

It is worth mentioning that the biochar physicochemical traits have a main effect on its efficiency. Indeed, biochar engineering, consisting of pyrolysis conditions and biomass composition, allows for achieving biochar traits that are optimal for specific conditions^52,53^. In the case of soil amendment, not all biochars are suitable for enhancing plant productivity. For example, the compound that is intended to be remediated should be smaller than the pore size of the biochar^54^.

Thus, the soil texture must be suitable for biochar application, which was clay loam with a basic pH in the present study (Table 1). Biochar application was more effective under dryland conditions (Fig. 5). Moreover, there was no significant difference between tillage systems in the first year. No tillage was more effective than minimum and conventional tillage on the grain yield in the second year. The use of biochar in no-tillage practices may have played a larger role in the rise in soil moisture levels during the second year, although it received less rainfall (305 mm) as compared to the first year (379 mm) (Figs. 1 and 5). Similarly, minimum tillage was more effective than conventional tillage. It seems that biochar is more effective in poor soil than in healthy and fertile soils. This result is in agreement with Zheng et al.^55^, who pointed out that biochar significantly enhanced maize yield in poor-nutrient soil compared to rich-nutrient soil. Biochar may play different role in other soils and in different climatic and irrigation conditions. Finally, when selecting biochar applications for each environment, it is important to consider the feedstock type, pyrolysis condition, and engineering technique^54^.

## Conclusion

*Nigella sativa* L. is a worthwhile herb that finds extensive usage in various industries. This research shows that the interaction effect of biochar and minimum tillage practices have the potential to significantly improve crop yields and grain oil content, irrespective of whether the land is well irrigated or not. Moreover, these improvements were comparable to those achieved by conventional tillage methods. It is worth noting that the fatty acid profile was affected by all the experimental factors as well as weather conditions. In the second year of the experiment, treatments involving minimum tillage and biochar were found to have the highest levels of linoleic acid. While the present study indicates that biochar and minimum tillage practices can deliver short-term benefits, it also provides compelling evidence of their long-term advantages, especially in dry and semi-dry regions.

These findings suggest that farmers can leverage these techniques to achieve sustainable increases in crop yields while minimizing their environmental footprints. By adopting these approaches, they can optimize their agricultural output while conserving precious resources for future generations.

## Data Availability

All data analysed during this study are included in this published article.
